# *CmVPS41* Is a General Gatekeeper for Resistance to *Cucumber Mosaic Virus* Phloem Entry in Melon

**DOI:** 10.3389/fpls.2019.01219

**Published:** 2019-10-01

**Authors:** Laura Pascual, Jinqiang Yan, Marta Pujol, Antonio J. Monforte, Belén Picó, Ana Montserrat Martín-Hernández

**Affiliations:** ^1^Centre for Research in Agricultural Genomics (CRAG) CSIC-IRTA-UAB-UB, C/Vall Moronta, Edifici CRAG, Bellaterra (Cerdanyola del Vallés), Barcelona, Spain; ^2^Institut de Recerca i Tecnologia Agroalimentàries (IRTA), Campus UAB, Bellaterra, Barcelona, Spain; ^3^Instituto de Biología Molecular y Celular de Plantas (IBMCP), Universitat Politècnica de València (UPV)-Consejo Superior de Investigaciones Científicas (CSIC), Valencia, Spain; ^4^COMAV, Institute for the Conservation and Breeding of Agricultural Biodiversity, Universitat Politècnica de València (UPV), Camino de Vera s/n, Valencia, Spain

**Keywords:** melon, genetic diversity, VPS41, *Cucumber mosaic virus*, Resistance, Phloem loading

## Abstract

Melon production is often compromised by viral diseases, which cannot be treated with chemicals. Therefore, the use of genetic resistances is the main strategy for generating crops resistant to viruses. Resistance to *Cucumber mosaic virus* (CMV) in melon is scarcely described in few accessions. Until recently, the only known resistant accessions were Freeman’s Cucumber and PI 161375, cultivar Songwhan Charmi (SC). Resistance to CMV in melon is recessive and generally oligogenic and quantitative. However, in SC, the resistance to CMV strains of subgroup II is monogenic, depending only on one gene, *cmv1*, which is able to stop CMV movement by restricting the virus to the bundle sheath cells and preventing a systemic infection. This restriction depends on the viral movement protein (MP). Chimeric viruses carrying the MP of subgroup II strains, like the strain LS (CMV-LS), are restricted in the bundle sheath cells, whereas those carrying MP from subgroup I, like the strain FNY (CMV-FNY), are able to overcome this restriction. *cmv1* encodes a vacuolar protein sorting 41 (CmVPS41), a protein involved in the transport of cargo proteins from the Golgi to the vacuole through late endosomes. We have analyzed the variability of the gene *CmVPS41* in a set of 52 melon accessions belonging to 15 melon groups, both from the spp *melo* and the spp *agrestis*. We have identified 16 different haplotypes, encoding 12 different CmVPS41 protein variants. Challenging members of all haplotypes with CMV-LS, we have identified nine new resistant accessions. The resistance correlates with the presence of two mutations, either L348R, previously found in the accession SC and present in other three melon genotypes, or G85E, present in Freeman’s Cucumber and found also in four additional melon genotypes. Moreover, the new resistant accessions belong to three different melon horticultural groups, Conomon, Makuwa, and Dudaim. In the new resistant accessions, the virus was able to replicate and move cell to cell, but was not able to reach the phloem. Therefore, resistance to phloem entry seems to be a general strategy in melon controlled by *CmVPS41*. Finally, the newly reported resistant accessions broaden the possibilities for the use of genetic resistances in new melon breeding strategies.

## Introduction

Melon (*Cucumis melo* L.) belongs to the family Cucurbitaceae and is one of the most productive crops in the world, with above 30 million tones/year (FAOSTAT http://www.fao.org/faostat/en/). *C. melo* has been traditionally divided into two subspecies defined by the pubescence of the ovary, spp. *melo* and spp. *agrestis* (Kirkbride, 1993), but most recent classifications use horticultural groups defined by vine, flowering, fruit traits, and geographic criteria. Pitrat (2017) described 19 horticultural groups including wild, feral, and domesticated melons: agrestis, kachri, chito, tibish, acidulus, momordica, conomon, makuwa, chinensis, flexuosus, chate, dudaim, chandalak, indicus, ameri, cassaba, ibericus, inodorus, and cantalupensis. These groups represent the broad phenotypical variability in agronomical traits, such as ripening, sugar accumulation, or fruit morphology displayed by the cultivars and landraces of this species. Most sources of resistance to viruses and pests identified so far belong to the acidulus and momordica groups from India and to the Far Eastern group of conomon, chinensis, and makuwa melons (those frequently referred to as conomon group) (Robinson and Decker-Walters, 1997; Blanca et al., 2012; Leida et al., 2015).

One of the most devastating plant viruses for melon is *Cucumber mosaic virus* (CMV), which produces typical mosaic in leaves and fruits and stunting plants. CMV is the type member of the *Cucumovirus* genus. It is a viral species with high sequence variability, resulting in large number of strains that can infect a broad range of plant species, including economically important crops, such as other main cucurbits (watermelon, cucumber, squash, and zucchini) as well as crops of the Solanaceae and Cruciferae families (Edwardson and Christie, 1991). On the basis of their sequence, CMV strains are divided into two subgroups [subgroup I (SG I) and subgroup II (SG II)] showing ∼70% sequence homology between groups (Roossinck, 2001). Genetic resistances are the most successful way of preventing viral infections. However, modern commercial cultivars usually lack genetic resistances, and it is necessary to introgress them from landraces and wild accessions (Pitrat, 2008; Pitrat, 2017). Until recently, only a few melon genotypes, mostly from Asia, have been reported as resistant to CMV. The most frequently resistance sources used in different studies have been the Japanese Freeman’s Cucumber (Karchi et al., 1975) and PI 161375, the Korean cultivar “Songwhan Charmi” (Con-SCKo) (from now on, SC) (Risser et al., 1977), classified as conomon and chinensis, respectively (Pitrat, 2017). Genetic studies show that, in both cases, resistance is oligogenic, recessive (Pitrat and Lecoq, 1980), quantitative (Dogimont et al., 2000), and also strain specific (Diaz et al., 2003). Other studies report resistance in several cultivars of the makuwa group (Pitrat and Lecoq, 1980; Hirai and Amemiya, 1989). Surveys carried out more recently have found other sources of resistance, mostly Indian cultivars of the momordica group but also some Iranian accessions (Dhillon et al., 2007; Fergany et al., 2011; Malik et al., 2014; Argyris et al., 2015). For most of them, the genetic control remains undetermined, and the strain specificity of these resistances was not reported. Therefore, the introduction of resistances to CMV in commercial cultivars is still challenging, and likely, the combination of genes/alleles from different sources would contribute to a broad-based resistance against these viruses.

The most studied resistance to CMV reported to date is that derived from the SC genotype. It is strain specific, recessive, and complex, controlled by at least three quantitative trait loci (QTLs) (Guiu-Aragonés et al., 2014). The major QTL is the gene *cmv1*, which by itself confers resistance against CMV strains of SG II (Essafi et al., 2009; Guiu-Aragonés et al., 2015). *cmv1* is also necessary for resistance to strains of SG I, but in this case, it is not sufficient and requires the contribution of the other two QTLs, as an example of the defense–counter defense established between pathogen and host (Guiu-Aragonés et al., 2014). The recessive resistance genes against viruses usually encode host proteins recruited by the virus to complete its cycle. Mutations in these genes may lead to resistance. Most recessive resistance genes identified encode either eukaryotic translation initiation factors (eIFs) or other factors involved in virus accumulation (for a review, see (Hashimoto et al., 2016). However, unlike previously reported recessive resistance genes, *cmv1* is involved in the transport of the virus and prevents systemic infection (Guiu-Aragonés et al., 2016). In the inoculated leaf of the resistant plant, the CMV strain LS (SG II) is able to move from cell to cell up to the vein but remains restricted to the bundle sheath (BS) cells and does not enter the phloem (Guiu-Aragonés et al., 2016). Therefore, the plant is resistant to systemic infection. However, the strain FNY (SG I) can overcome this barrier and enter the phloem. The viral protein that determines if the virus is transported to the phloem is the movement protein (MP). A CMV carrying the MP of LS cannot be transported to the phloem when inoculated into a plant carrying *cmv1*. However, a CMV clone carrying the MP from FNY will be translocated (Guiu-Aragonés et al., 2015). *cmv1*, therefore, is a gatekeeper that determines if the virus is either transported to the phloem to produce a systemic infection (susceptible plant) or remains restricted to the BS cells (resistant plant). This discrimination occurs depending on the MP and only in the BS cells, since *cmv1* does not affect the cell-to-cell movement in other cells of the inoculated leaves.

Map-based cloning of *cmv1* has shown that this gene encodes a vacuolar protein sorting 41 (CmVPS41) (Giner et al., 2017). This protein is normally involved in the transport of proteins in vesicles from the late Golgi to the vacuole as part of the “homotypic fusion and vacuole protein sorting” complex (Asensio et al., 2013; Pols et al., 2013). CMV might, therefore, recruit CmVPS41 through its MP to be transported to the phloem. The polymorphism L348R, present in SC, was reported as the causal mutation that restricts phloem entry in this accession. (Giner et al., 2017).

Here, we explore the possibilities of *CmVPS41* as target for resistance by sequencing it in 50 new melon genotypes. We have selected the accessions to represent 15 different horticultural groups covering an ample spectrum of melon diversity. We report 14 new haplotypes and 9 newly discovered resistant accessions, including Freeman’s Cucumber. The resistance to CMV shown by these genotypes was demonstrated to be allelic with that of SC. Moreover, the resistance is manifested as a restriction in phloem entry, as it occurs in the previously reported SC genotype (Guiu-Aragonés et al., 2016). These new alleles represent new opportunities for diversifying the resistance to CMV derived from *CmVPS41* during breeding programs.

## Materials and Methods

### Plant and Virus Material

Melon genotypes used were a Spanish “Piel de Sapo” inbred line (In-PsSp) (from now on PS), traditionally included in the inodorus group and now classified within the ibericus group (Pitrat, 2017), the Korean accession PI 161375, cultivar Songwhan Charmi (Con-SCKo) (from now on SC), belonging to the chinensis group, and 50 additional accessions listed in **Table 1**. These accessions represent many of the groups described by Pitrat 2017: cantalupensis, ameri, chandalak, dudaim, chate, flexuosus, conomon, makuwa, chinensis, momordica, acidulus, tibish, chito, and agrestis. We included at least one accession per group and included a higher number of accessions from the groups where resistances had been previously described (conomon, makuwa, chinensis). Most of the selected accessions were molecularly characterized previously (Leida et al., 2015) and classified in seven structure groups. The selection included accessions from all the structure groups detected, thus representing a wide range of melon diversity (**Table 1**; **Supplementary Table 1**).

**Table 1:** Accessions analyzed in this work. Indicated are CmVPS41 gene haplotype, CmVPS41 protein allele, response to the inoculation with CMV-LS number of accessions tested, accessions code, assigned group according to previous structure analysis.

Haplotype gene	Protein type (12)	CMV-LS*	Accessions**	Structure group***
Hap-1	Prot-1	S (2/4)	Ib-PSSP, Am-Bol, Can-VedFran, Con-YaPuJa	3, A, 1, (1,6)
Hap-2	Prot-2	S (8/26)	26 accessions****	5, (7,6), (6,2), A, (6,5), 6, (6,3), (6,7,2), (5,4)
Hap-2-C1012Irak	Prot-2-C1012Irak	S	Dud-C1012Irak	–
Hap-2-Ksud	Prot-2	–	Tibish-Ksud	7
Hap-2-QPMAfg	Prot-2	R	Dud-QPMAfg	6,7,2
Hap-3	Prot-3	R (4/4)	Con-SCKo, Con-GMJa, Con-Chi52Chi, Con-MielChi	6, (1,6), (6,5)
Hap-4	Prot-4	R (4/4)	Con-FreeCJa, Con-NanChi, Con-OgonChi, Con-ShiroJa	6
Hap-4-Pat81Ko	Prot-4-Pat81Ko	R	Con-Pat81Ko	6
Hap-5	Prot-5	S (2/2)	Flex-AryaInd, Ag-TriInd	A
Hap-5-Kakru	Prot-5	S	Ag-KakInd	–
Hap-5-TGR1551Zimb	Prot-5 TGR1551Zimb	S	Ac-TGR1551Zimb	7,6
Hap-6	Prot-2	–	Ag-15591Gha, Ag-TayCam	7
Hap-6-C38Nig	Prot-6-C38Nig	S	Ag-C38Nig	7
Hap-6-C836CV	Prot-6-C836CV	S	Ag-C836CV	–
Hap-7-CarBItA	Prot-7-CarBIta	S	Chate-CarBIta	1,4,5
Hap-7-SanIlPhil	Prot-7-SanIlPhil	S	Con-SanIlPhil	6,7

*Response to LS inoculation. S, susceptible; R, resistant. In parentheses, number of accessions tested out of total number of accessions with a given haplotype.

**First part of accession code indicates melon type, last part country of origin.

***Structure groups according to Leida et al. (2015): 1 and 2: Cantalupensis accessions, French charentais and reticulatus, respectively, 3: Spanish inodorus, 4: Worldwide inodorus and ameri, 5: Asian ameri, 6: Oriental melons, conomon, chinensis, and makuwa, 7: African agrestis, A: Admixture, accessions that could not be associated to any of the groups.

****Con-LongtChi, Con-KNMJa, Mom-KhaInd, Dud-DudaimFra, Con-CUM206Chi, Mom-PI124Ind, Chi-VellInd, Dud-QAPMSwitz, Mom-PI414Ind, Con-GouChi, Con-Baish1Chin, Con-Baish2Chin, Con-LongtChi, Con_Xiao1Chin, Con-HerChin, Am-KizilUzbe, Con-BaishChin, Con-Co6Chi, Con-GapPhi, Con-PauPol, Con-XiaoChi, Dud-DudaimAfg, Mom-MR1Ind, Ag-WCHInd, Am-ChanRus, Con-OmGMJa.

CMV strain LS, belonging to SG II and provided by Dr. J. Diaz-Pendón (CSIC, EELM, Málaga, Spain), was used for inoculations.

### Sequencing *CmVPS41*, Analysis, and Identification of Haplotypes and Proteins.

*CmVPS41* genomic sequence (http://melonomics.cragenomica.es/) was used to design seven primers pairs in order to amplify the complete coding sequence (Giner et al., 2017), using Primer3 software (Untergasser et al., 2012) (http://bioinfo.ut.ee/primer3-0.4.0/) (**Supplementary Table 2**). PCR amplified fragments were purified with sepharose columns and sequenced by capillary electrophoresis at Macrogen (Macrogen Europe, Amsterdam, The Netherlands). Sequences were analyzed with Sequencher® version 5.0 sequence analysis software (Gene Codes Corporation, Ann Arbor, MI, USA (http://www.genecodes.com) and aligned with PS exons in order to detect polymorphisms. The obtained information was used to reconstruct *CmVPS41* complete coding sequence for each accession and to define haplotypes. Nucleotide sequences for each identified haplotype were translated with ExPASy translation tool (http://web.expasy.org/translate/). DNA or protein sequences were aligned using ClustalW (Thompson et al., 1994). Phylogenic trees from the nucleotide alignment were calculated employing neighbor-joining method, with Jukes–Center distance and 100 bootstraps. For proteins, we employed neighbor-joining method, with Tamura distance and 100 bootstraps. For the *CmVPS41* gene, haplotype networks were constructed using PopArt (Leigh and Bryant, 2015) employing the minimum spanning method (Bandelt et al., 1999) Effects of the polymorphisms in the CmVPS41 protein were predicted with Protein Variation Effect Analyzer (PROVEAN) [http://provean.jcvi.org/index.php, (Choi et al., 2012)] changes were considered deleterious when the predicted PROVEAN score was lower than -2.5. The Plaza database, version 3.0 dicots (http://bioinformatics.psb.ugent.be/plaza/), was used to identify orthologues of the *VPS41* gene in other plant species whose genome was available.

### Inoculation With CMV And Virus Detection

Inoculation and virus detection were done as previously described by Essafi and collaborators (2009), using at least six plants by tested genotype. Briefly, seeds were pregerminated by soaking them in water overnight and then kept for 2–4 days in 12 h light at 28°C. Seedlings were grown in growth chambers (Sanyo MLR-350H, Sanyo Electric Biomedical Co, Osaka, Japan) during the whole essay, in long-day conditions of 22°C for 16 h with 5,000 lx of light and 18°C for 8 h in the dark. Viral inocula were prepared from freshly symptomatic leaves of zucchini (“Chapin F1,” Semillas Fito SA, Barcelona) and rub inoculated onto the cotyledons of young melon plants still without leaves. Symptoms were scored visually at 7, 14, and 21 days postinoculation (dpi), and viral detection was done by double antibody sandwich ELISA in young developed leaves from the six inoculated plants. Analysis was done with CMV-specific polyclonal antisera (Loewe Biochemica GmbH, Otterfing, Germany) following manufacturer’s protocol. ELISA reactions were measured spectrophotometrically at 405 nm using the VICTOR3 V multilabel plate reader (PerkinElmer). ELISA was considered positive when absorbance at 405 nm was larger than twice the negative control value. PS and SC were used as susceptible and resistant controls, respectively. SC12-1-99 NIL, derived from the NIL SC12-1 (Essafi et al., 2009), carrying a shorter introgression of SC that contains the *cmv1* gene, was also used as resistant control. Negative controls were prepared from leaf tissue extracts of mock-inoculated plants.

### Crosses And Allelism Tests

A subset of the resistant accessions identified (at least one by haplotype) was crossed with melon accessions PS and NIL SC12-1-99. At least six F1 plants from each cross were tested against CMV-LS. After inoculation with CMV-LS, a resistance source was considered allelic only when none of the F1 plants from the cross with SC-12-1-99 plants showed symptoms, and the F1 plants from the cross with PS were infected.

### Detection Of CMV-LS In The Phloem

First true leaf of six plants per genotype was inoculated with CMV-LS. After detection of yellow areas around the entrance points, the petiole of this leaf was collected at 11 dpi. RNA was isolated, and the presence of the virus in the phloem was tested by reverse transcription PCR as described (Guiu-Aragonés et al., 2015). CMV-LS, specific primer LS1-1400R (GAAGCATTCCACATATCGTAC), was used for RT, and the same primer together with LS1-900F (GTTTTATTTACAAGAGCGTACG) were used to amplify a 500-bp fragment of the viral genome.

## Results

### Identification Of New CmVPS41 Melon Haplotypes And Protein Variants.

To study the genetic variability of the melon VPS41gene, 52 melon genotypes, including PS and SC, from 15 different melon groups were chosen. The *CmVPS41* genomic sequence was either sequenced or was obtained from the available whole genome sequences (Sanseverino et al., 2015) [those from the two controls, PS and SC, and from one cantalupensis melon, Can-VedFran, one dudaim melon, Dud-C1012Irak, and one wild African *agrestis*, Ag-C836CV (**Table 1**)].

**Figure 1:**
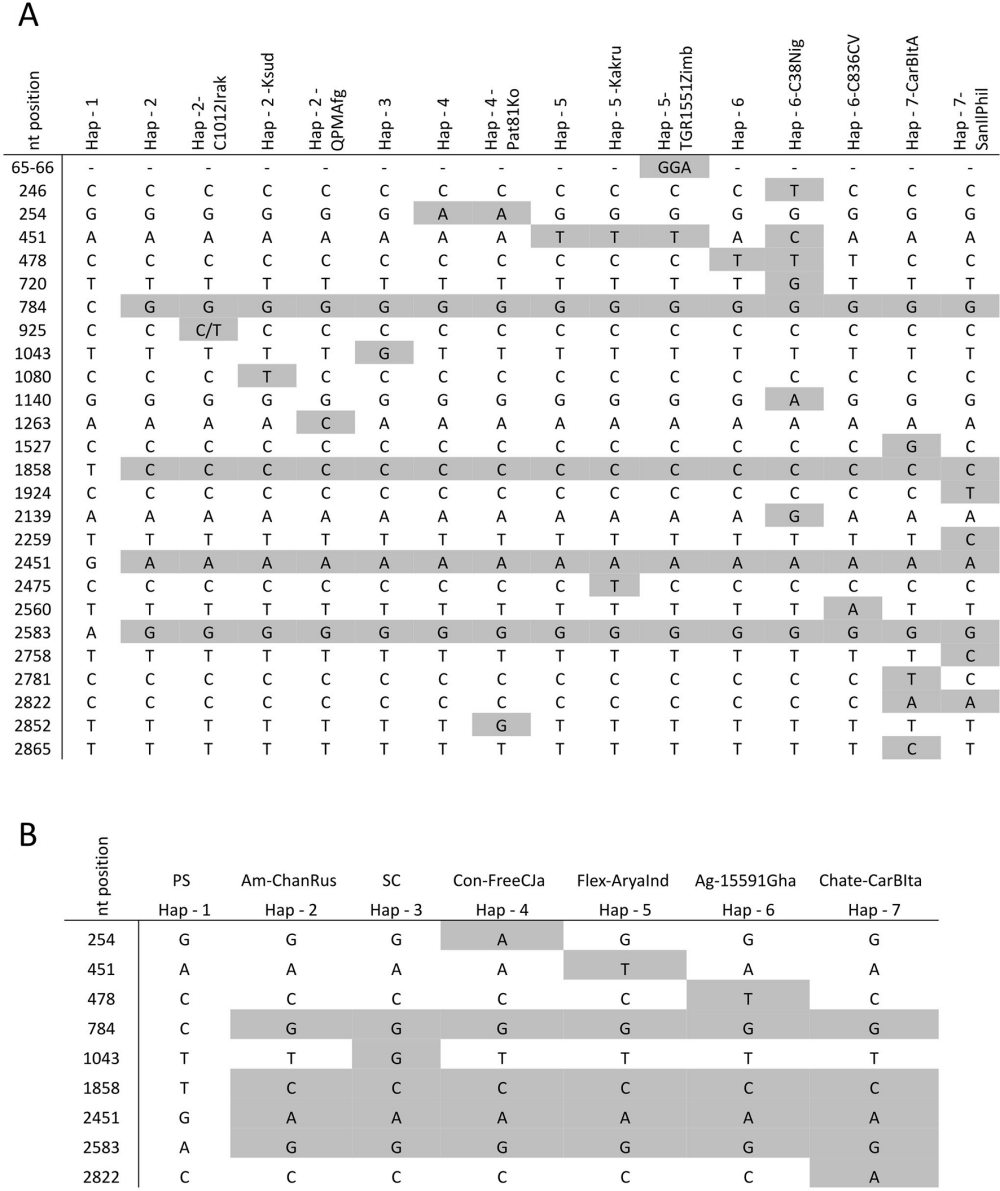
Haplotypes and frequency observed in the *CmVPS41* gene from 52 melon genotypes. **(A)** Nucleotide polymorphisms present in the melon haplotypes analyzed. In gray, the polymorphisms found in the sequenced accessions. **(B)** Core haplotypes excluding singletons. nt: nucleotide number.

**Figure 2:**
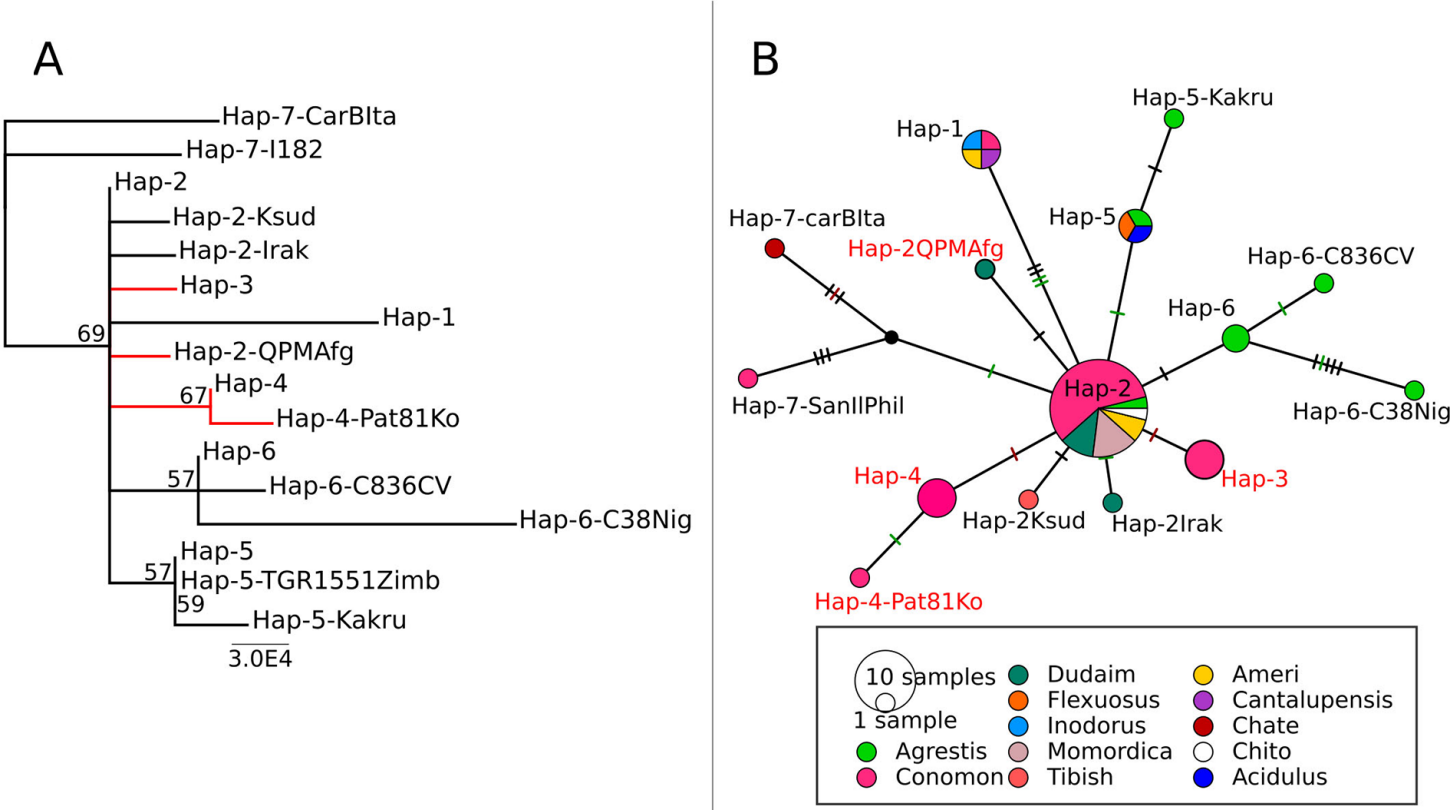
**(A)** *CmVPS41* phylogenetic tree of the different haplotypes. Branches where all the accessions showing a given haplotype are resistant are marked in red. **(B)** Reconstruction of haplotypes evolution in a network. Polymorphisms are indicated as vertical lines, black synonymous SNPs. Green, amino acid change with neutral effect according to the PROVEAN score. Red, amino acid change with deleterious effect. Indels are not analyzed. Each circle represents a different haplotype, and circle size is proportional to the number of accessions carrying the haplotype. Circles are colored by melon type. Haplotypes were all the accessions are resistant are written in red.

In the whole set of 52 accessions, 27 single nucleotide polymorphisms (SNPs) were identified (**Figure 1**), being 18 of them singletons and defining 14 new *CmVPS41* haplotypes (**Figure 1**), different from those from PS (Hap-1) and SC (Hap-3) (**Figure 2**). The most represented haplotype (Hap-2) was present in 26 melon genotypes (**Table 1**, **Figure 2B**). After removing the singletons, seven core haplotypes remained, and two haplotype blocks were evident, one from position 1 to 1,858 and another from 1,858 to 2,583 (**Table 1**, **Figure 1B**).

The haplotype 1 (Hap-1) was present in the two modern European cultivars, PS (the susceptible control) and Védrantais (Can-VedFran), representing the Spanish inodorus and French cantalupensis groups, respectively (**Table 1**). Hap-1 was also shared by two cultivars: the Japanese Yamato Purinsu (Con-YaPuJa), classified as makuwa, but grouped within the cantalupensis group by Leida et al. (2015), and the American landrace Am-Bol, from Bolivia, which also showed genome admixture in previous analysis (**Table 1**).

The haplotype 2 (Hap-2) was present in the vast majority of accessions, mostly from Asia, belonging to different horticultural types, such as the assayed Indian momordica, the chandalack and ameri accessions from Central Asia, and most of the makuwa and chinensis Far eastern melons (all in the well-defined structure group of oriental melons) (**Table 1**). Asian dudaim melons (molecularly related to the oriental group, but with admixture from other groups) also showed this haplotype, although two of the dudaim accessions from the Near East had unique Hap-2 derived haplotypes (Dud-C1012Irak and Dud-QPMAfg). Hap-2 was also present in two small-fruited accessions from India, the chito-type Velleri (Chi-VellInd) and the wild agrestis (Ag-WChInd). The only African type with Hap-2 was the Tibish accession Tibish-KSud, considered a melon domesticated in Africa from wild African agrestis (Endl et al., 2018) (**Table 1**, **Figure 2B**).

The other five haplotypes likely arose by single nucleotide mutations from haplotype 2 (**Figure 2B**) mostly in positions between 254 and 1,043, while no mutation was observed between positions 1,858 to 2,583, suggesting that the SNPs in this part of the sequence are in high linkage disequilibrium, whereas SNPs in the first part of the gene are in linkage equilibrium.

Interestingly, two of these five haplotypes, haplotypes 3 (Hap-3, that of the resistant control SC) and 4 (Hap-4), were present in the remaining accessions of the oriental melon group from China, Korea, and Japan (conomon, chinensis, and makuwa) that belong to the same structure group than Far Eastern melons displaying Hap-2 (**Table 1**).

Haplotype 5 (Hap-5) was found in two wild Indian agrestis, trigonus and kakru (Ag-TriInd and Ag-KakInd), and in the unique accessions analyzed of the flexuosus (Flex-AryaInd) and acidulus (Ac-TGR1551Zimb) groups, whereas haplotype 6 (Hap-6) was characteristic of the wild Central African agrestis accessions (**Table 1**). The last haplotype (Hap-7) was found in two accessions, one Italian (Chate-CarBIta) and one chinensis type from the Philippines (Con-SanIlPhi) (**Table 1**).

### The New CmVPS41 Haplotypes Produce 10 New Variants.

**Figure 3:**
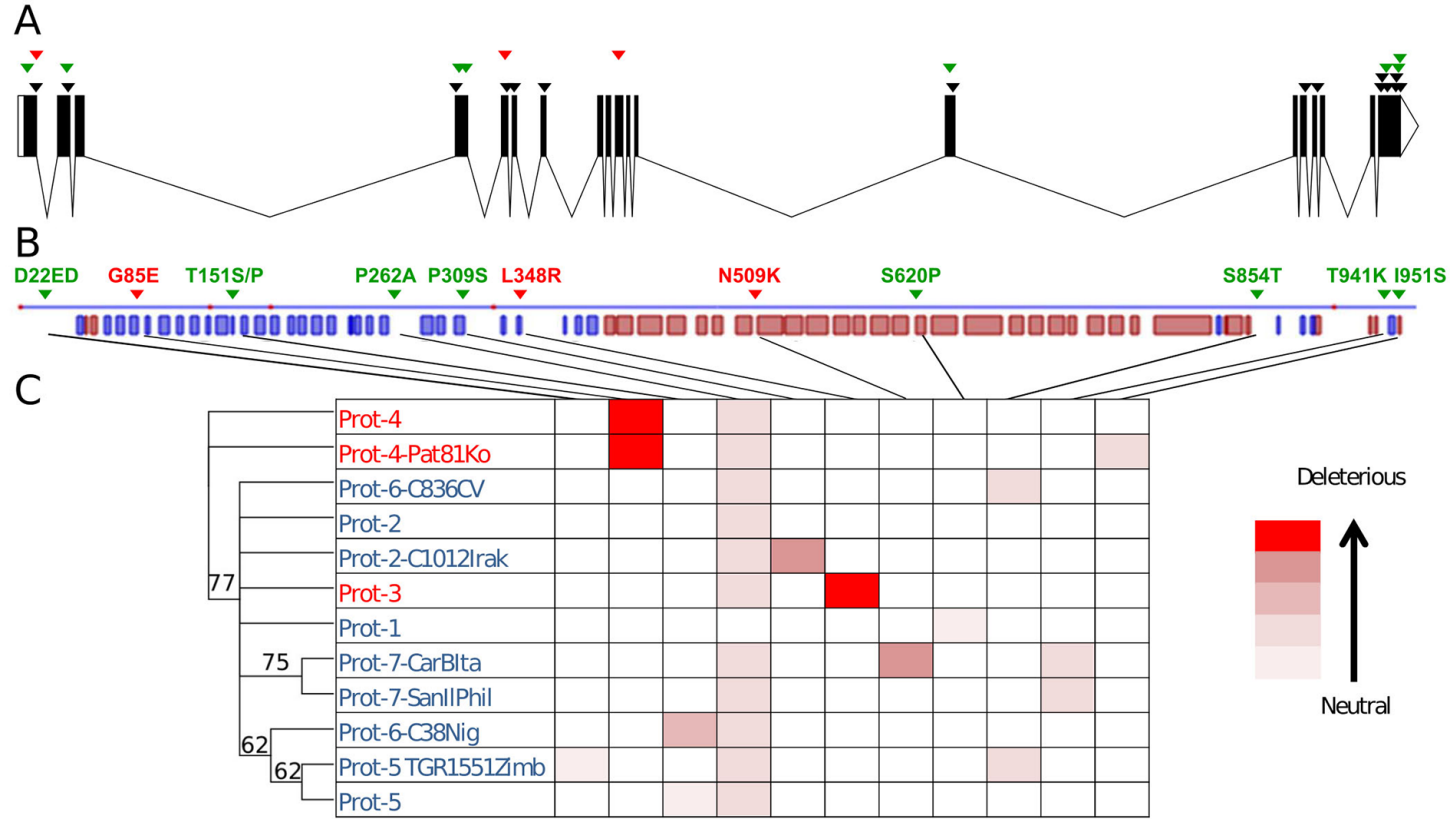
Polymorphism in *CmVPS41*. **(A)** *CmVPS41* genomic sequence. Boxes are exons. Polymorphisms with respect to In-PsSp *CmVPS41* are marked. Black, synonymous substitutions. Green, amino acid change with neutral effect. Red, amino acid change with deleterious effect. **(B)** Secondary structure of CmVPS41 protein from PS. In blue, *β* sheets; in red, α helix. All amino acid changes are indicated. Green, change with neutral effect. Red, change with deleterious effect. **(C)** Phylogenetic tree of the different protein alleles. In red, resistant genotypes. In blue, susceptible genotypes. The relative effect of their amino acid changes is shown by a gradient of red color, from neutral to deleterious, according to the values given in **Table 2**.

Out of the 27 polymorphisms found (including the two changes in position 451), 15 produced synonymous substitutions and one added an amino acid. The 11 nonsynonymous substitutions found produced amino acid changes with different effects on the final protein (**Table 2**, **Figure 3**). Eight of those substitutions have effects catalogued as neutral for the function of the protein. However, two of them had a strong theoretical deleterious effect, the G85E substitution, with a PROVEAN score of -7.008, and the L348R substitution, with a PROVEAN score of -5.929, whereas the D509K had a weak, almost neutral effect of -2.857.

**Table 2:** Description of the amino acid changes predicted in the CmVPS41 protein. The effect according to Protein Variation Effect Analyzer (PROVEAN) is indicated.

nt position	Hap1 codon	Alternative	aa change	Shift value	Effect
**65-66**	GAC	GAGGAC	D22ED	3.231	Neutral
**254**	GGG	GAG	G85E	-7,008	Deleterious
**451**	ACT	TCT/CCT	T151S/T151P	0,889/-1.852	Neutral/neutral
**784**	CCA	GCA	P262A	-0,903	Neutral
**925**	CCT	TCT	P309S	-2,254	Neutral
**1,043**	CUA	CGA	L348R	-5,929	Deleterious
**1,527**	AAC	AAG	N509K	-2.857	Deleterious
**1,858**	TCA	CCA	S620P	1,203	Neutral
**2,560**	TCT	ACT	S854T	-0,464	Neutral
**2,822**	ACA	AAA	T941K	-0,545	Neutral
**2,852**	ATT	AGT	I951S	-0,328	Neutral

The L348R substitution is the previously reported in SC and identified as the causal mutation of the resistance to CMV-LS exhibited by this genotype (Giner et al., 2017). It is also present in other three genotypes (those sharing the Hap-3), all belonging to the makuwa and chinensis groups: the Japanese cultivar Ginsen makuwa (Con-GMJa), and the Chinese cultivars Miel Blanc (Con-MielChi) and China51 (Con-Chi51Chi) (**Table 1**). The latter was classified within the French cantalupensis group by Leida et al. (2015), but showed a high degree of admixture with the oriental melons group to which the other cultivars belong. The substitution G85E has not been previously reported and is present in five Far Eastern accessions, belonging to the conomon, makuwa, and chinensis groups (oriental melons structure group), which share Hap-4. Two of the cultivars showing this mutation are the conomon Japanese cultivars Freeman’s Cucumber (Con-FreeCJa) and Shiro uri Okayama (Con-ShiroJa), as well as two Chinese makuwa [Nanbukin (Con-NanChi) and Ogon9 (Con-OgonChi)] and one Korean chinensis (Pat 81 (Con-Pat81Ko). The accessions sharing the same amino-acid sequence either in Hap-3 or in Hap-4 represent true different accessions, as they show clear phenotypic and molecular polymorphism (Leida et al., 2015; **Supplementary Figure 1**). Therefore, the haplotype was fixed before the diversification of these cultivars.

Our results indicate that the most common *CmVPS41* haplotype (Hap-2) is not restricted to specific melon groups, being present in highly diverse Asian accessions from different origins and representing different horticultural groups. However, some less frequent haplotypes only occur or are more frequent in specific groups, such as Hap-3 and Hap-4 in oriental melons and Hap-5 and Hap-6 in wild Indian and African agrestis, respectively.

### Only Melon Genotypes Carrying CmVPS41 Variants With Strong PROVEAN Score Are Resistant To CMV-LS

Twenty-nine melon genotypes belonging to all melon groups and showing different *CmVPS41* haplotypes were challenged with CMV-LS to assess their resistance to this strain. As shown in **Table 1**, out of the 29 genotypes inoculated, 10 were resistant to CMV-LS. Nine of them carry the amino acid substitutions L348R (Ginseng makuwa, China 51, Miel Blanc, as well as the previously described SC) or G85E (Freeman’s Cucumber, Shirouri Okayama, Pat81, Nanbukin, and Ogon9), whereas none of the genotypes carrying other amino acid substitutions correlated with resistance to CMV-LS. L348R and G85E are the polymorphisms showing the strongest PROVEAN score (**Figure 3**).

The role of L348R substitution on the resistance found in SC has previously been demonstrated (Giner et al., 2017). Therefore, this substitution should also be responsible for the resistance of the three new carrier genotypes. Regarding the substitution G85E, reported here for the first time, it is shared by Pat81 and the other four genotypes with Hap-4, Freeman’s Cucumber, Shiro Uri Okayama, Nanbukin, and Ogon 9. Pat81 carries an additional acid change with respect to PS, I951S, that shows a very weak PROVEAN score on the protein. As the mutation G85E is shared with Freeman’s Cucumber and the other three resistant genotypes, which lack any other mutation, it is reasonable to think that the polymorphism responsible for resistance in Pat81 is G85E, whereas I951S has no effect on the phenotype. The substitution N509K reveals a deleterious, but almost neutral score, and indeed, the only accession carrying it, an Italian Landrace belonging to the ancient group of chate melons (Sabato et al., 2019) (Chate-CarBIta, haplotype 7-CarBItA), is susceptible to CMV-LS (**Table 1**, **Figure 3**). Therefore, these observations suggest that only those amino acid variations with a strong PROVEAN score lead to resistance to CMV-LS.

Interestingly, in the assayed collection, the dudaim cultivar Queen’s pocket melon (Dud-QPMAfg) (Hap- 2) do not present any amino acid change with a strong effect on the protein and is, nevertheless, resistant to CMV-LS. QPM presents the most frequent amino acid sequence. Out of the 29 genotypes analyzed, showing this amino acid sequence, 9 of them were tested for resistance to CMV-LS, and only this dudaim cultivar was resistant to CMV-LS (**Table 1**), which may indicate that either the resistance from this accession has another genetic control or the phenotype is due to any other change, linked to 1,263 nucleotide synonymous mutation, not included in the current report.

Therefore, we have characterized the variability of the gene *CmVPS41* in 50 new genotypes, 9 of them resistant to CMV-LS which belong to 4 melon botanical groups: Ginsen makuwa, China 51, Nanbukin and Ogon (makuwa), Miel blanc and Pat 81 (chinensis), Freeman’s Cucumber and Shiro Uri Okayama (conomon), and Queen’s pocket melon (dudaim), which could be used in breeding programs, depending on the phenotype or the need for additional resistances.

### Allelism Tests

To confirm that *CmVPS41* was responsible for the resistant phenotype in the newly reported melon accessions, plants from Ginsen makuwa, Pat81, Freeman’s Cucumber, and Queen’s pocket melon were crossed with the near isogenic line SC12-1-99, which contains a small introgression from SC carrying the *CmVPS41* gene (Hap-3), in the PS background (Essafi et al., 2009). The same accessions were crossed with the susceptible genotype PS as control. All PS F1 hybrid plants were infected, confirming that the resistance in these accessions is recessive, as it is in SC (Pitrat and Lecoq, 1980; Guiu-Aragonés et al., 2014). However, each F1 progeny from crosses with SC12-1-99 was resistant to CMV-LS systemic infection. Given that the resistance is recessive, this result confirms that in all tested accessions, it is controlled by the same gene. Interestingly, the F1 from Queen’s pocket melon x SC12-1-99 is also resistant to CMV-LS. Therefore, it is allelic also in this genotype, which implies that *CmVPS41* from Queen’s pocket melon is responsible for the resistance to CMV-LS despite its predicted protein (Prot-2) being equal to other susceptible accessions (**Table 1**).

### The New Resistant Genotypes Restrict CMV-LS Phloem Entry

**Figure 4:**
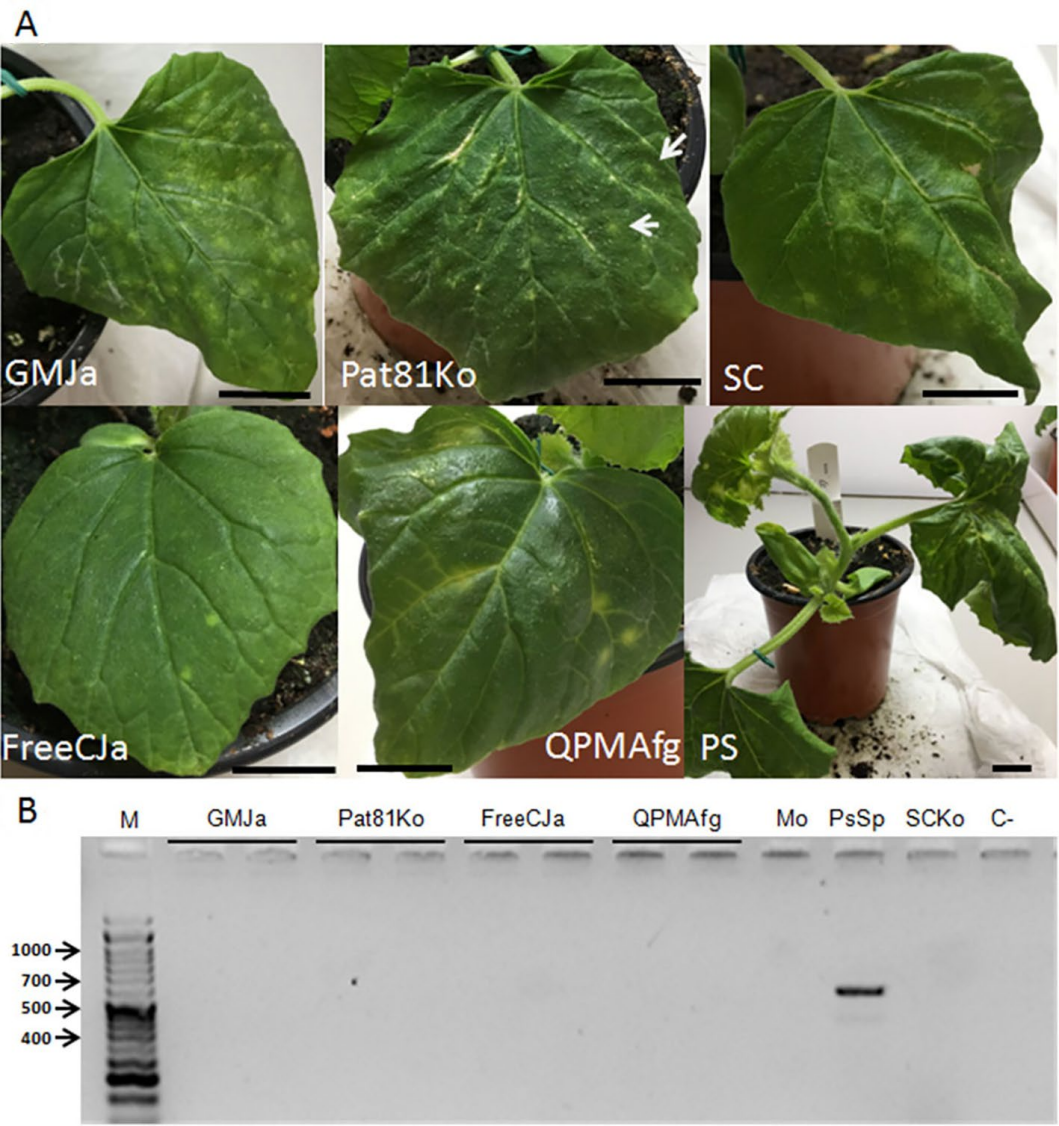
Resistance to CMV-LS in exotic melon genotypes. **(A)** Inoculated leaves from resistant melon genotypes at 11 dpi showing the halos where infection initiates. Black scale bars indicate 2 cm. In PS genotype, at 11 dpi, the whole plant is infected. **(B)** Reverse transcription PCR (RT-PCR) of petiole samples from inoculated leaves of two individual plants per genotype. Mo: Mock inoculated plant. C-: negative control for PCR. M: size marker. On the left, sizes of relevant bands of the marker, in base pairs.

Resistance to CMV-LS in the accession SC acts at the level of viral phloem entry (Guiu-Aragonés et al., 2016), whereas the virus can replicate and move cell to cell. To test if in the new resistant accessions CMV-LS was able to invade the phloem, the first true leaf of plants from accessions Ginsen makuwa, Pat81, Freeman’s Cucumber, and Queen’s pocket melon was inoculated with CMV-LS. After 11 dpi, all accessions had developed yellow areas around the entrance points in the inoculated leaf, indicating that the virus was able to replicate and move cell to cell in these accessions (**Figure 4A**), as had been previously reported for SC (Guiu-Aragonés et al., 2016). However, none of the accessions had developed symptoms of CMV infection except PS, used as positive control. Then, the petiole of the inoculated leaf was collected, and the presence of the virus in the phloem was tested by reverse transcription PCR. As shown in **Figure 4B**, the virus was absent from the phloem of all resistant accessions, including the resistant control SC, whereas in PS, the virus had reached the phloem and could be detected in the petiole of the inoculated leaf. This indicated that the resistance to CMV-LS in the four tested accessions was acting at the level of phloem entry, as is also the case of SC, where the virus is restricted in the BS cells of the minor veins (Guiu-Aragonés et al., 2016). Therefore, restriction to phloem entry seems to be a common strategy for recessive resistance to CMV in melon.

## Discussion

We have explored the polymorphism of the gene *CmVPS41* in 52 melon genotypes belonging to a representation of most major horticultural groups reported in the species (Pitrat, 2017). In total, 16 haplotypes have been observed, including PS and SC, leading to 12 different protein variants, including those already described from PS and SC (Giner et al., 2017). VPS41 is a protein involved in intracellular trafficking of proteins and vesicles from late Golgi to the vacuole that seems to have an essential role in the cell (Niihama et al., 2009) Attempts to overexpress *CmVPS41* in melon under the 35S promoter were unsuccessful (L. Pascual, unpublished), whereas expression from its own promoter produced fully viable transgenic plants (Giner et al., 2017), suggesting that CmVPS41 protein does not admit neither strong changes in its structure nor in its expression levels. In fact, out of the 12 polymorphisms leading to amino acid changes, only two of them, G85E and L348R, highly conserved residues located in conserved regions (**Supplementary Figure 2**, Giner et al., 2017), have a theoretical strong effect on the protein, and both of them correlate with resistance to CMV-LS. No VPS41 structure has been resolved in any species, and thus, we cannot model the influence of these amino acid changes in the protein structure. However, secondary structure prediction using PROFAcc (https://www.predictprotein.org/) shows that there are two regions in the protein. From amino acid 42 to 399, there is a region with only ß sheets, whereas from residue 405 to 825, there are only α helices. Both amino acid 85 and 348 reside in the ß sheets region, and both lay in loops between two ß sheets (**Figure 3B**). As only subtle changes are allowed in VPS41 protein, it suggests that changes in loops are more easily allowed than others in more structured regions. These loops might lay in the areas involved in the interaction with the viral MP, the determinant of virulence (Guiu-Aragonés et al., 2015), so that these changes would affect only the viral infection without affecting the vital function of transporting cargo proteins to the vacuole. Interestingly, the two parts of the protein nearly correspond to the two parts observed in the gene, since up to nucleotide 1,828, there are more fixed SNPs than from nucleotide 1,829 to the 3′ end of the gene. This way, the first half of the gene, which appears to have admitted more mutations, corresponds to the ß-sheet structure and is where the causal mutations reside. The second part of the gene, which is in high linkage disequilibrium, corresponds to the α-helix region. It is possible that this second part of the gene is under stronger selection than the 5′ part.

Melon was likely domesticated twice: in Africa and Asia (Endl et al., 2018) where wild accessions are frequently found. Different *CmVPS41* haplotypes have been identified in wild types from different origins, while most of the analyzed cultivated accessions shared Hap-2. India, and specifically the highly variable momordica group, is considered the origin of most Asian landraces and of the widely commercialized cultivar groups (Blanca et al., 2012; Leida et al., 2015). *CmVPS41* seems to be uniform within the momordica group. The Hap-2, found in all momordica accessions, is also present in ameri, dudaim, and a large set of oriental melons. However, the Far Eastern melons (accessions of the conomon/chinensis/makuwa groups from Japan, China, and Korea), characterized by their low molecular diversity (Blanca et al., 2012; Esteras et al., 2013; Leida et al., 2015), are highly variable for this gene, including accessions carrying three different haplotypes. Two of them, Hap-3 and Hap-4, lead to CMV-LS resistance. These Far Eastern cultivars were originated after the diversification of melon from India towards Far East Asia (Gonzalo et al., 2019); therefore, the mutations related to resistance either could arise in the early diversification of these groups or represent ancestral variability that has been maintained likely by selection. Apart from the previously studied SC, the Japanese cultivars Freeman’s Cucumber (Con-FreeCJa), Shiro uri Okayama (Con-ShiroJa), Ginsen makuwa (Con-GMJa), and the Chinese cultivar China51 (Con-Chi51Chi) were previously reported as resistant to CMV (Karchi et al., 1975; Pitrat and Lecoq, 1980; Provvidenti, 1998), although the resistance was not studied in detail. The remaining accessions are new sources of resistance to CMV, and some of them, such as Ogon 9 (Con-OgonChi) and Pat 81 (Con-Pat81Ko), have been previously used in melon breeding for their resistance to fungus (Perchepied and Pitrat, 2004; Roig et al., 2012).

The Hap-1, which leads to CMV susceptibility, is found in accessions representing not only the main European commercial types, inodorus and cantalupensis, but also in South American and Asian landraces. Therefore, the new resistant accessions sharing the previously characterized SC haplotype and the new ones having the newly described haplotype characterized by a new deleterious mutation are interesting materials for breeding commercial melons against CMV.

Another interesting source of resistance is the dudaim-type Queen’s pocket melon, which does not present any putative causal mutation, showing the same protein found in other 25 susceptible accessions. However, Queen’s pocket melon is resistant to CMV-LS, and moreover, the resistance is allelic with that of SC as assessed by crossings with the resistant NIL SC12-1-99. Dudaim melons are morphological and molecularly different from other melon groups (Gonzalo et al., 2019). The resistance observed here could be due to an independent mutation in a noncoding sequence not covered in the current report. For example, it could be due to an alternative splicing which would produce a slightly different protein still able to keep its role in vacuole trafficking. Alternatively, the silent mutation at position 1,263 could have some effect in the RNA folding, with a role in the regulation of the protein. Further work is needed to address if the mutation is located either in a noncoding regulatory sequence of *CmVPS41* or in another tightly linked gene and to understand the events underlying the resistance to CMV-LS in QPM genotype. However, the later possibility is very unlikely, since, given the recessive nature of the resistance, it would mean that, in SC, the other gene would bear also a mutation leading to resistance to CMV-LS, so that, in the F1, the mutation would be in homozygosis. However, in that case, the putative mutation should already be in homozygosis in SC and would have been responsible for the resistance in that genotype. As map-based cloning demonstrated, the only gene responsible for the resistance to CMV-LS in SC was *CmVPS41* (Giner et al., 2017).

VPS41-mediated resistance to CMV in the new resistant genotypes restricts CMV phloem entry, to finally impede the development of a systemic infection. Guiu-Aragonés et al, (2016) reported that, in the resistant introgression line SC12-1-99 (carrying SC resistance gene), the virus movement is blocked in the BS cells that surround the vein, and therefore, it does not reach the phloem to produce a systemic infection. Therefore, our results with the new resistant genotypes again support the role of *CmVPS41* in this resistance. Moreover, the fact that the new resistant accessions belong to four different melon groups suggests that restriction to systemic infection *via* restriction of phloem entry as mechanism for resistance to CMV-LS appeared very early during the melon domestication or diversification. The role of *CmVPS41* in resistance to CMV, and its generality in resistant melon accessions opens new possibilities for using different breeding approaches based on genes that control different steps of the viral cycle, to promote resistances to different viruses. Targeting eIFs genes has provided a useful tool against several viruses in melon (Rodríguez-Hernández et al., 2012; Chandrasekaran et al., 2016) and in other species (Sanfaçon, 2015). A strategy based on producing melon plants pyramiding mutations in eIFs, which would target viral translation/replication, and in VPS41, targeting viral movement, would speed up breeding strategies for sustainable plant protection against viruses of different families.

## Data Availability Statement

All datasets generated for this study are included in the manuscript/**Supplementary Files**.

## Author Contributions

LP designed and carried out experiments and contributed to write the manuscript. JY did experimental work, MP supervised experimental work, and AM and BP contributed to write the manuscript. AM-H designed experiments and wrote the manuscript.

## Funding

AM-H was supported by the grants AGL2012-40130-C02-01 and AGL2015-64625-C2-1-R from the Spanish Ministry of Economy and Competitiviness (cofunded by FEDER funds) and by the CERCA Proframme/Generalitat de Catalunya. We acknowledge financial support from the Spanish Ministry of Economy and Competitiveness, through the “Severo Ochoa Programme for Centres of Excellence in R&D” 2016-2019 (SEV‐2015‐0533). AM was supported by grant AGL2015-64625-C2-2-R from the Spanish Ministry of Economy and Competitivity. Grants AGL2017-85563-C2-1-R by the Spanish Ministery of Science, Innovation and Universities (cofunded with FEDER funds) and by the PROMETEO project 2017/078 (to promote excellence groups) by the Conselleria d’Educació, Investigació, Cultura i Esports (Generalitat Valenciana) were supporting BP. JY was supported by a China Scholarship Council (CSC) fellowship.

## Conflict of Interest

The authors declare that the research was conducted in the absence of any commercial or financial relationships that could be construed as a potential conflict of interest.
