# Supplementary material for: CmVPS41 Is a General Gatekeeper for Resistance to Cucumber Mosaic Virus Phloem Entry in Melon
Source: Front Plant Sci. 2019 Oct 1;10:1219. doi: 10.3389/fpls.2019.01219 (PMC6781857; doi:10.3389/fpls.2019.01219)

Supplementary figure 1. Accessions resistant to CMV-LS. A Accessions of the oriental melon group sharing Haplotype 3 with the resistant control SC. **B** Accesions of the oriental melon group sharing Haplotype 4. **C** Resistant accession of the dudaim group with Hap-2-QPMAfg.

A

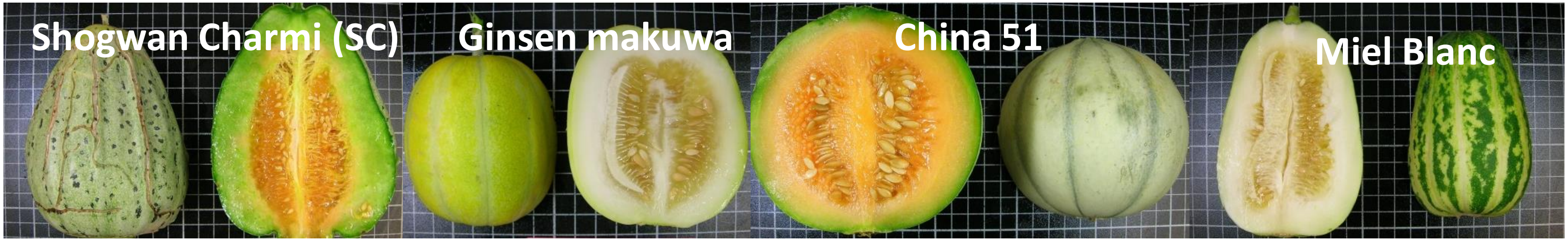

B

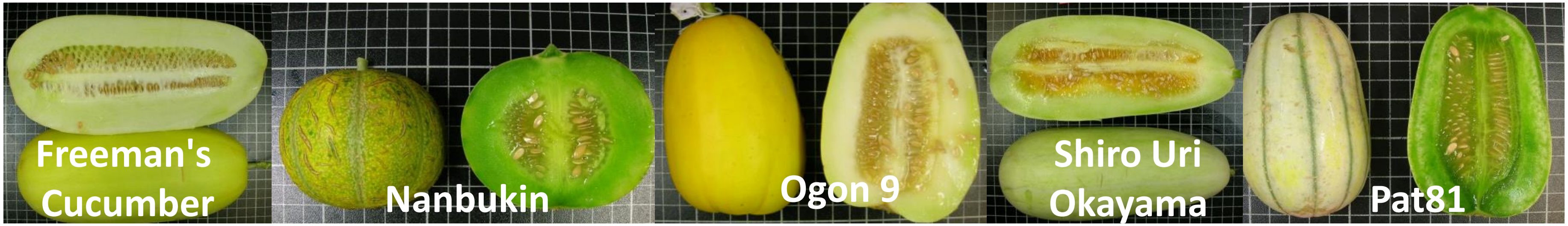

C

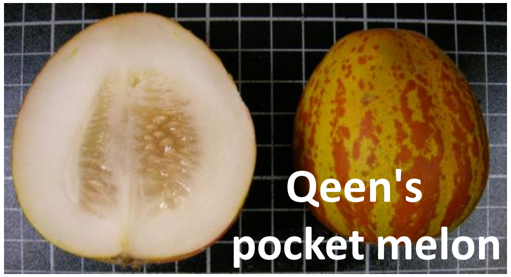

Supplement: Supplementary file 1 [file DataSheet_1.pdf]
