# Supplementary material for: CmVPS41 Is a General Gatekeeper for Resistance to Cucumber Mosaic Virus Phloem Entry in Melon
Source: Front Plant Sci. 2019 Oct 1;10:1219. doi: 10.3389/fpls.2019.01219 (PMC6781857; doi:10.3389/fpls.2019.01219)
Supplement: Supplementary file 2 [file DataSheet_2.pdf]

Supplementary figure 2. Amino acid sequence of the CMVPS41 fragment flanking the G85E change detected in protein variant Prot4. The sequence was aligned against the corresponding sequence from representative plant species and melon varieties. Marked in red is the nonsynonymous mutation detected.

```
C. melo  GMJa              LASDAASCLAVAERMIALGTHAGTVHILDFLGNQVKEFPAHTAVVNDLSFDTEGEYVGSC
         Pat81Ko           LASDAASCLAVAERMIALGTHAGTVHILDFLENQVKEFPAHTAVVNDLSFDTEGEYVGSC
         SC                LASDAASCLAVAERMIALGTHAGTVHILDFLGNQVKEFPAHTAVVNDLSFDTEGEYVGSC
         FreeCJa           LASDAASCLAVAERMIALGTHAGTVHILDFLENQVKEFPAHTAVVNDLSFDTEGEYVGSC
         QPMAfg            LASDAASCLAVAERMIALGTHAGTVHILDFLGNQVKEFPAHTAVVNDLSFDTEGEYVGSC
         PS                LASDAASCLAVAERMIALGTHAGTVHILDFLGNQVKEFPAHTAVVNDLSFDTEGEYVGSC
C. Sativus                 LASDAASCLAVAERMIALGTHAGTVHILDFLGNQVKEFPAHTAVVNDLSFDTEGEYVGSC
A. thaliana                LSNDAASCIAVAARMIALGTHDGTVRILDLLGNQVKEFRAHTAPVNDINFDTEGEYIGSC
S. lycopersicum            LSADAATCIAVAERMIALGTHGGAVHILDFLGNQVKEFAAHTAAVNDLCFDTDGEYVGSC
G. max                     LASDAASCIAVAERMIALGTHGGTVHILDFLGNQVKEFSAHASVVNDLSFDTEGEYIGSC
V. vinifera                LSSDAACCIAIAERMIALGTHDGTVHILDLLGNQVKEFRAHNATVNDLSFDVEGEYIGSC
R. communis                LSNDAASCIAVAERMIALGTLDGTVHILDFLGNQVKEFAAHTAAVNDLSFDIEGEYIGSC
                           *: *** *:*:* *******  *:*:***:* ****** ** : ***: ** :***:***
```
