# Supplementary material for: CmVPS41 Is a General Gatekeeper for Resistance to Cucumber Mosaic Virus Phloem Entry in Melon
Source: Front Plant Sci. 2019 Oct 1;10:1219. doi: 10.3389/fpls.2019.01219 (PMC6781857; doi:10.3389/fpls.2019.01219)
Supplement: Supplementary file 4 [file Table_2.docx]

| **Supplementary table 2.** Primers used to amplify *CmVPS41* exons | | |  |  |
| --- | --- | --- | --- | --- |
|  |  |  |  |  |
|  | **Primer** | **Sequence** | **Tm** |  |
| Exon 1-3 | YJQ 1F | CCCCATAATTGATGTCACCAC | 55ºC |  |
|  | YJQ 1R | CAGCTAGTTGCAGTAGCATCC |  |  |
| Exon 4-6 | pAG 046 F | CTTTTTAGTTTTGATAGGGCATGT | 55ºC |  |
|  | pAG 046 R | CCACAAAAGCGACATTCTA |  |  |
| Exon7 | YJQ 5F | GGCAGGTCTTACAAGTCCCAA | 55ºC |  |
|  | YJQ 5R | TGAGATGTCCAAGCGTGTCT |  |  |
| Exons 8-12 | pAG 044 F | GTCACTTTTGTATGAGGTTAGTAT | 55ºC |  |
|  | pAG 044 R | CCACAAAAGCGACATTCTA |  |  |
| Exon 13 | pAG 048 F | CGGTTATGATTCTGTTTAGCTTGA | 55ºC |  |
|  | pAG 048 R | AGACTACATGGGGCGGCTAC |  |  |
| Exons 14-17 | YJQ 3F | CGCAGAATTAACATGCCAAG | 55ºC |  |
|  | YJQ 3R | GTGAAAGGGAACCACAGTCA |  |  |
| Exons 18,19 | YJQ 4F | GTTGCGACGAGTCATGAAAA | 55ºC |  |
|  | LP16 REV | CTTGCCACCTTTTTCAGTGTT |  |  |
|  |  |  |  |  |
